# Transient Single Cell Hypoxia Induced by Localized Galvanostatic Oxygen Challenge

**DOI:** 10.1021/acsmeasuresciau.4c00100

**Published:** 2025-04-01

**Authors:** Marlene H. Hill, Gabriel N. Meloni, Bruno G. Frenguelli, Patrick R. Unwin

**Affiliations:** † Department of Chemistry, University of Warwick, Coventry CV4 7AL, U.K.; ‡ Molecular Analytical Science Centre for Doctoral Training (MAS CDT), University of Warwick, Coventry CV4 7AL, U.K.; § School of Life Sciences, University of Warwick, Coventry CV4 7AL, U.K.; ∥ Institute of Chemistry, Department of Fundamental Chemistry, University of São Paulo, São Paulo, São Paulo 05508-000, Brazil

**Keywords:** hypoxia, microelectrode, scanning electrochemical microscopy, confocal microscopy, fluorescence, single cell measurements

## Abstract

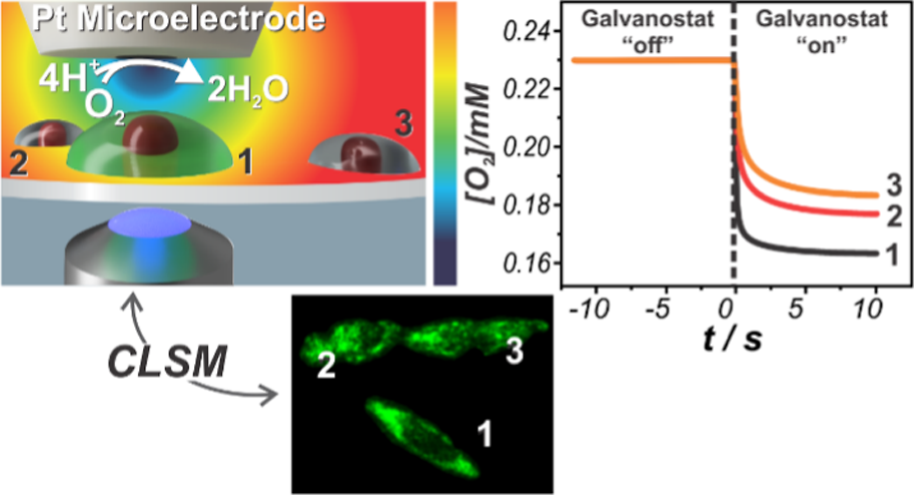

Studying cells exposed to low and controllable oxygen levels is key to investigating various fundamental aspects of pathological states, such as stroke and cancer. At present, available methodologies applied in vitro focus on large groups of cells exposed to low oxygen conditions through slow-time approaches, such as environmental incubators or microfluidic devices. Here, we demonstrate a novel approach for titrating the local oxygen concentration around individual adhered PC12 cells, enabling single cells within a population to be exposed to hypoxic-like conditions. A 25 μm diameter platinum disk microelectrode performing the oxygen reduction reaction (ORR) at constant current (galvanostatic control) is used as a microscale oxygen scavenger that can be positioned precisely over individual cells. By coupling the galvanostatic oxygen challenge with confocal laser scanning microscopy (CLSM) and a commercially available hypoxia dye (Image-iT Green hypoxia reagent), we monitor the response of single cells when exposed to depleted oxygen concentrations over time. Numerical simulations are used to characterize the oxygen and pH gradient imposed by the microelectrode at different cathodic currents, revealing that within seconds, the oxygen depletion zone reaches a steady-state condition, extending a few microelectrode radii into solution, while the corresponding pH gradient is strongly compressed by the buffer solution. Cells under the microelectrode show a marked increase in average fluorescence rate relative to control, reporting their hypoxic conditions and demonstrating the effectiveness of the proposed method. Heterogenous cell response in a challenged group is also observed, highlighting the ability of this approach to investigate the natural heterogeneity in cell populations. This work provides a platform and roadmap for future studies of cellular systems where the ability to control and vary oxygen concentration on a rapid time scale would be beneficial.

## 1 Introduction

Extracellular and intracellular oxygen (O_2_) levels are fundamental for the physiological function and survival of aerobic species, playing a vital role in cellular bioenergetics^1^ and metabolic reactions.^2^ Oxygen-sensing feedback systems in aerobic organisms detect and quickly respond to changes in O_2_ levels.^3^ Atmospheric oxygen levels are ∼21% (normoxia) at sea level,^4−6^ but can drop to approximately one-third of that at extreme altitudes, such as the summit of Mount Everest (8848 m above sea level).^7^ Hypoxia, a term used for abnormally low levels of oxygen in tissues and cells,^8^ can trigger several biochemical processes to maintain oxygen homeostasis and promote survival. When these processes are heavily disrupted, however, various complex conditions can ensue, such as cancer and tumors,^8,9^ cardiac ischemia and stroke,^10^ sleep apnea,^11^ Alzheimer’s disease,^12^ and inflammatory diseases.^13^ Most tissue and cell types in vivo are exposed to oxygen levels varying between 1 and 6% (physioxia),^14,15^ much lower than normal atmospheric levels. Tissue and cells cultured in vitro and without a vascularization system, however, are typically exposed closer to normal atmospheric oxygen levels, which could be considered hyperoxic.^5^ Despite this, hypoxia studies in vitro, where cells are exposed to lower than atmospheric oxygen levels, are performed extensively and are still relevant, serving as a proxy for in vivo hypoxia conditions.

Assays performed in vitro usually involve gassing the cell culture medium with a gas mixture that contains varying levels of oxygen, nitrogen, and carbon dioxide. An environmental chamber or incubator is typically set at 5% CO_2_, while the O_2_ levels equilibrate at ∼18%, subjecting the entire cell culture medium to a uniform and lower than ambient oxygen level.^6,14^ With these procedures, it can take several hours for the cell culture medium to equilibrate with the gas mixture. Microfluidic devices can modify O_2_ levels around small cell populations or even single cells^16^ within minutes.^17^ Although faster than large cell population assays, there are still significant temporal resolution limits to which the cell response to hypoxic conditions can be studied. The fast onset of hypoxic conditions would allow the investigation of more dynamic cellular events and could be applied to study acute, transient oxygen deprivation, akin to cells and tissues subject to ischemic events.^10^

Microelectrodes have been used to monitor the concentration of species around individual cells with high temporal resolution.^18−23^ Microelectrodes have also been used to measure oxygen levels around cells and tissues using the oxygen reduction reaction (ORR) current recorded at a platinum surface and generally considering the four-electron direct pathway for oxygen reduction.^24−31^ In these studies, the oxygen depletion zone generated by the electrode extends a few microelectrode radii into the solution,^32−35^ changing local and intracellular oxygen levels.^27^

Herein, we employ a platinum microelectrode to control local oxygen levels dynamically via galvanostatic control of ORR rates and subject adherent PC12 cells to lower oxygen concentrations in a highly controlled manner. We coupled the local electrochemical oxygen challenge with confocal laser scanning microscopy (CLSM), using an indirect and irreversible fluorescent oxygen probe, to visualize and investigate the time response of individual cells when challenged in this way. Numerical simulations were used to calculate and predict the local oxygen concentration around the challenged cell, and its neighbors, providing a spatiotemporal view of the oxygen concentration profiles during the galvanostatic oxygen challenge. By addressing individual cells, we bridge the gap between cell population and single cell measurements, gaining individual entity (cellular) information without the solution and species confinement concerns of single cell studies with microfluidic devices.

## 2 Experimental Section

### 2.1 Chemicals

All solutions were prepared using deionized water (Milli-Q, resistivity ca. 18.2 MΩ cm at 25 °C). Image-iT Green hypoxia reagent (Invitrogen, Thermo Fisher Scientific), horse serum (Sigma-Aldrich-H1270, HS), fetal bovine serum (Sigma-Aldrich-F7524, FBS), penicillin–streptomycin (Sigma-Aldrich-P4333), dimethyl sulfoxide (Sigma-Aldrich, DMSO), Live Cell Imaging Solution (Invitrogen), were used as received and without further purification. The stock hypoxia reagent solution (mM) was prepared by adding 10 μL of DMSO to the Image-iT Green hypoxia reagent vial, which was kept for multiple experiments. The stock solution was diluted to a final concentration of 10 μM with F12K (Kaighn’s modification, Gibco) media and used for staining cell cultures prior to the experiments.

### 2.2 PC12 Cell Culture Conditions and Sample Preparation

Adherent PC12 cells (Addex-Bio) were grown in F12K media, supplemented with 2.5% FBS, 15% HS, and 1% ampicillin/streptomycin, according to the supplier’s protocol, and kept in an incubator at 37 °C and 5% CO_2_ atmosphere. Cells were split at ∼70–80% confluency, checked with an optical microscope (as shown in Figure S1),^36^ plated in small Petri dishes (50 mm diameter, WillCo) at ∼30–50% confluency, and placed back in the incubator to adhere overnight.^37^ After 20 h, Image-iT Green hypoxia solution (10 μM) was added to the cell dish for 1 h to stain cells. The cell media was then removed and replaced with the Live Cell Imaging solution. The Live Cell Imaging Solution is based on a HEPES buffer instead of bicarbonate, as in the F12K media, allowing experiments to be performed in normal atmospheric conditions. Detailed composition of the Live Cell Imaging Solution can be found in Table S1. Adherent PC12 cells exhibiting a polygonal shape were selected for the galvanostatic oxygen challenge and image analysis.^38^

### 2.3 Confocal Laser Scanning Microscopy Settings

Imaging of PC12 cells stained with Image-iT Green hypoxia dye was carried out using an inverted Leica TCS SP5 X CLSM (Leica, Germany). Cells were imaged using an argon laser, excitation wavelength 488 nm. Emission was recorded between 515 and 525 nm. All images were collected using a 20× oil immersion objective (NA = 1.4) with the confocal pinhole set to 90.05 μm. The *z*-stack time-lapse images were captured in 512 × 512 pixel images at a 1 kHz scan rate (ca. 500 ms per image). The *z*-slice thickness was 0.84 μm, and five slices, capturing the middle to the base region of the cells, were recorded for all *z*-stacks captured in the experiments. A 30 s interval was employed between every time-lapse frame (a *z*-stack). Images were captured synchronously with the electrochemical data.

### 2.4 Galvanostatic Electrochemical Experiments

**Figure 1:**
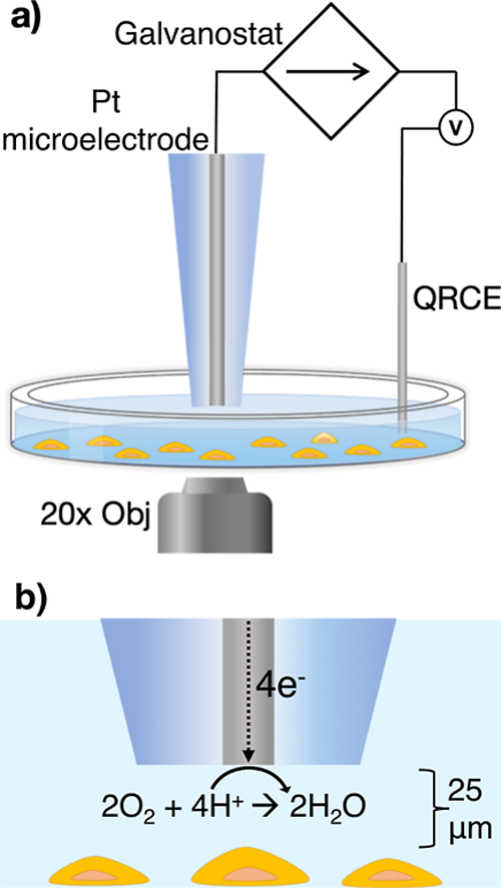
Experimental setup for single cell oxygen challenge, showing (a) the Pt microelectrode, QCRE, CLSM objective, and the PC12 cell culture stained with fluorescent hypoxia marker. (b) Zoomed-in illustration of the microelectrode-cell gap, showing the microelectrode-cell separation (working distance) and ORR reaction at the Pt microelectrode.

All electrochemical experiments were performed using a custom-made galvanostat operating in a 2-electrode setup. A 25 μm diameter platinum disk microelectrode with a total outer diameter (including the glass body) of 350 μm was used as the working electrode (Supporting Information, SI-2). The microelectrode was fabricated according to a procedure reported previously.^39^ A chloridized silver wire (Ag/AgCl) was used as a quasi-reference counter electrode (QRCE). A 3-axis manual micropositioner (M-462, Newport, US) was used to position the microelectrode above target cells for the oxygen challenge experiments. The microelectrode-cell separation was assessed optically using a manual focus control drive integrated into the CLSM instrumentation, enabling *z*-axis adjustment. The tip-substrate distance was set to 25 ± 0.5 μm for all experiments, derived from the difference between the *z*-height focal planes of the microelectrode surface and the upper cell surface. This was possible due to the high axial resolution of the CLSM setup.^40^ A schematic of the experimental setup is given in Figure 1a. The oxygen challenge was performed by reducing oxygen at the microelectrode at a constant current. The upper limit for the galvanostatic ORR current was set below the diffusion-limited value, calculated by simulations (see below and Supporting Information Section SI-3), to avoid any side reactions, such as solvent breakdown or production of peroxy species.^41,42^

### 2.5 Image Analysis

ImageJ software^43^ was used to quantify the change in Image-iT Green hypoxia reagent fluorescence intensity of whole adherent PC12 cells in response to the electrochemical O_2_ challenge. At every time point in the time-lapse, the entire fluorescence value of a *z*-stack was collapsed to a single image by adding all fluorescence values within the stack. The outline of the cells in the image was masked, and the average intensity values of the masked area were calculated. Normalized fluorescence was calculated by dividing the entire time-lapse by the intensity value of the first frame at the onset of the electrochemical O_2_ challenge. The rate of fluorescence change was calculated by a linear fit of the fluorescence value over time.

### 2.6 Statistical Analysis

All reported rates of fluorescence change values are the average, plus or minus the standard deviation (±SD), of 19 individual cells. Data sets were analyzed using OriginPro 2019b software; a paired *t* test was performed to compare control and challenged data sets. *P*-value < 0.05 was considered significant. The variance between the control and challenged data sets was compared by an *F*-test.

### 2.7 Numerical Simulations

Numerical simulations were performed using the finite element method (FEM) in COMSOL Multiphysics version 5.6. A 3D simulation domain was used to represent the microelectrode geometry and the cells near the electrode, approximated to spherical caps with a diameter equal to the largest cell dimension. The 3D environment allowed for multiple cells to be simulated, capturing not only the target cell directly under the microelectrode but also its neighbors. As ORR consumes protons, a set of further simulations was also performed in a 2D axisymmetric cylindrical geometry to evaluate the effect on local pH of the different galvanostatic currents used in the Live Cell Imaging Solution. More details regarding both types of simulations are provided in the Supporting Information, Section SI-3.

## 3 Results and Discussion

### 3.1 Understanding the Galvanostatic Oxygen Challenge

**Figure 2:**
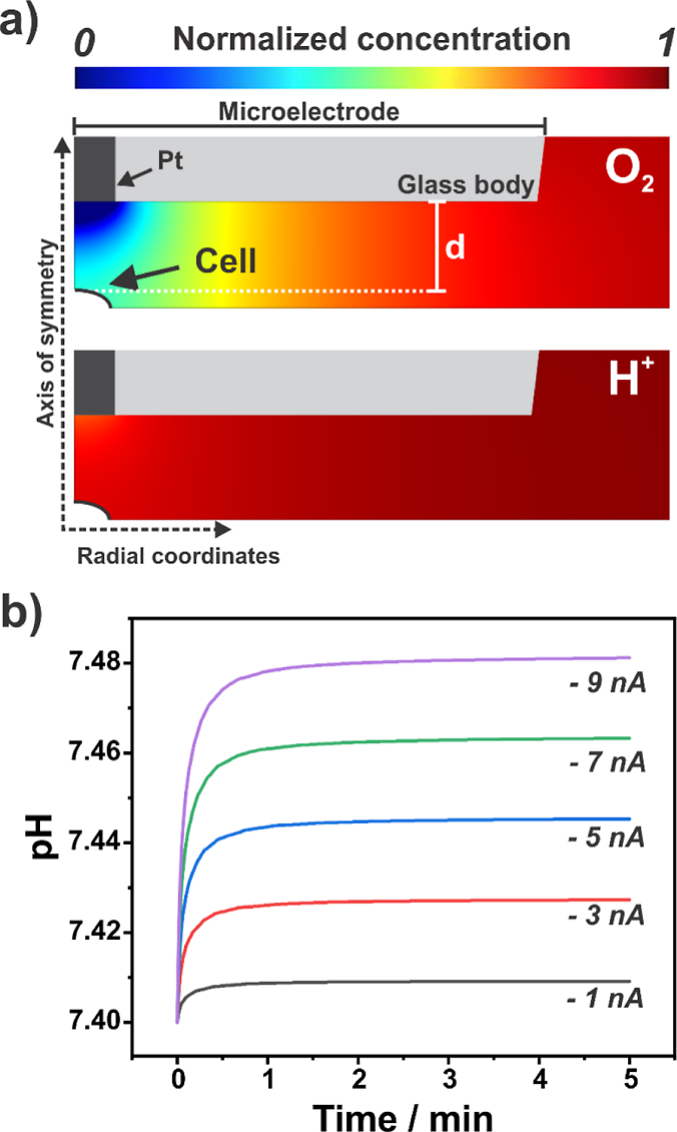
(a) Simulated oxygen (top) and pH (bottom) profiles in the gap between a 25 μm diameter microelectrode positioned 25 μm from a cell (working distance—“d”) after 5 min performing ORR at an applied current of −7 nA. (b) The effect of microelectrode ORR currents (−1, −3, −5, −7, and −9 nA) on the average pH at the targeted cell surface as a function of time after applying the galvanostatic challenge to the microelectrode.

FEM simulations were first used to determine the largest possible ORR current at the chosen microelectrode-cell separation of 25 μm (“d” in Figure 2a). The diffusion-limited current was calculated by integrating the oxygen flux at the microelectrode/solution interface when a concentration boundary of 0 mol L^–1^ of O_2_ was set at the electrode surface and assuming a four-electron, direct path for oxygen reduction at platinum.^30^ An oxygen flux equivalent to a cellular respiration rate of 2.15 pmol s^–1^, an average value for PC12 cells,^18^ was applied to the cell outline, setting the initial local oxygen concentration in the microelectrode-cell gap before the microelectrode ORR oxygen flux was applied. Under these conditions, a diffusion-limited oxygen current of −9.52 nA was found and set as the upper limit, not to be exceeded for the galvanostatic O_2_ challenge.

Figure 2a shows the normalized concentration profiles for O_2_ and H^+^ around the microelectrode and a target cell in an axisymmetric cylindrical geometry when an ORR current of −7 nA was simulated. The normalization for the profiles is the spatial concentration divided by the bulk concentration. The oxygen depletion zone is localized but is far more extensive than the pH change, as the latter is minimized by the buffer capacity of the media. Figure 2b shows the impact of different ORR currents on the average pH values at the cell membrane. The extent of the pH change was dependent on the ORR current, with larger values resulting in larger pH swings. At the extreme value of −9 nA, a change of 0.08 units is seen. This is considered a small pH challenge to most mammalian cell lines^44^ and lies within the optimal growth pH of the PC12 cell line used here (7.4–7.6). Also, such variation in pH is commonly found within many cell culture media.^45^ We conclude that under in vitro physiological conditions, the electrochemical challenge is effectively selective toward O_2_.

**Figure 3:**
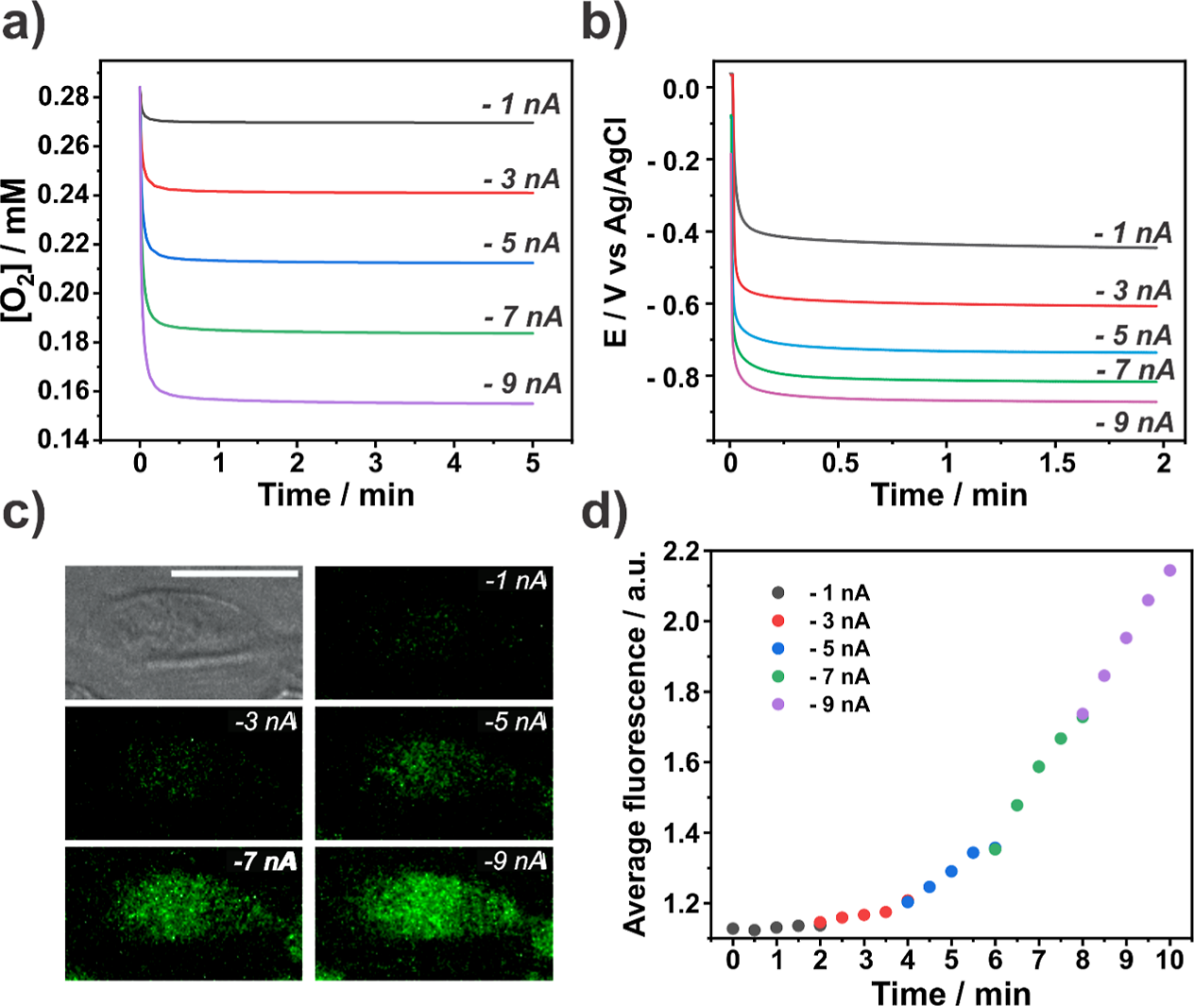
(a) Simulated oxygen concentration profile over time at the substrate surface under the microelectrode tip for different ORR currents. (b) Experimental galvanostatic potential over time for different ORR currents with the microelectrode placed directly over a single cell. (c) Brightfield image of a single cell before the oxygen challenge and fluorescence images of the same cell, stained with hypoxia dye, recorded after 2 min of galvanostatic challenge at different ORR currents. (d) Average fluorescence intensity over time for one target cell shown in panel (c). Scale bar in panel (c) is 25 μm.

For all simulated ORR currents, the oxygen concentration value at the cell reaches a steady-state value quickly (<60 s), as shown in Figure 3a, much faster than bulk methods and single-cell microfluidic devices.^16^ Moreover, the galvanostatic challenge allows for fine control of the oxygen levels over the cell, with larger ORR currents resulting in lower local O_2_ concentrations. We note that if the challenge were to be performed in a potentiostatic mode, biofouling of the electrode, typically seen in measurements in biological media,^31,46,47^ would be a concern, reducing the oxygen consumption flux. The galvanostatic approach avoided this issue by setting the ORR current; the oxygen reduction rate at the microelectrode is fixed, with the galvanostat adjusting the applied potential as needed. Hence, the oxygen flux at the electrode surface is constant over time. This is seen in Figure 3b, which shows the potential applied by the galvanostat over time for different ORR currents during an oxygen challenge experiment over a single cell. For all currents, the potential shifts slightly toward more cathodic values, over time: at longer times to compensate for electrode deactivation; and the decreasing local oxygen concentration which changes substantially in the initial few seconds. By remaining within the diffusion-limited ORR rate (vide supra), the potential never reaches extreme cathodic values (<−1.0 V) associated with solvent breakdown and the production of peroxy species fluxes.^36,48^

This experiment was performed over a single PC12 cell, labeled with the hypoxia dye (Figure 3c), with the ORR current starting from −1 nA and increasing stepwise to a final value of −9 nA, with every challenge lasting 2 min. The Image-iT Green hypoxia reagent fluoresces upon enzymatic cleavage by nitroreductase as its expression is upregulated under low oxygen conditions.^49,50^ This process involves an irreversible chemical transformation, leading to a sustained fluorescence signal even after oxygen levels return to normal levels.^51^ As an end point marker, this reagent can be used to visualize the impact of the different ORR currents on the intracellular O_2_ levels based on fluorescent readouts (Figure 3d).

Although the FEM model predicts a steady-state oxygen concentration around the cell within 30–60 s (Figure 3a), the fluorescence intensity increases linearly with time and increases with current (Figure 3d). The continuous change in fluorescence value could be attributed to adaptive cellular mechanisms actively “resisting” the challenge or the indirect nature of the hypoxia dye measurement. In particular, possible cellular mechanisms triggered in lower oxygen conditions include activation of hypoxia-inducible factor (HIF) pathways, metabolic adaptions, and programmed cell death processes, such as apoptosis.^52,53^ To investigate whether the oxygen challenge could induce apoptosis, a viability assay employing the cell death indicator, propidium iodide (PI),^54^ was carried out on a target cell exposed to a galvanostatic challenge at −7 nA (Supporting Information section SI-3). The lack of PI fluorescence (Figure S3a) indicates that the target cell was still alive after the challenge.

### 3.2 Oxygen Challenge of Cells

We now focus on studying the effect of the oxygen challenge across multiple cells. We selected an ORR current of −7 nA, as it demonstrated a significant impact on oxygen concentration (Figure 3a), i.e., a marked change in fluorescence intensity (Figure 3d) and a minimal impact on the pH around the target cell (Figure 2a). As the fluorescence values change linearly over time (vide supra) and all cells are subjected to the same ORR current (−7 nA), we focus on studying the rate of fluorescence change of each cell with time as a proxy for the rate of intracellular O_2_ lost by the cell. We hypothesize that if all conditions (experimental parameters and cell metabolism and health) remain the same, the rate of change of fluorescence for the challenged cells should be similar. Various cells (*n* = 19) across four dishes, positioned directly below the same Pt microelectrode at the same working distance (25 μm), were the target for the oxygen challenge for the same total duration (5 min). Before the galvanostatic challenge started, the fluorescence of the cells was measured for 5 min. These fluorescence data were used as the baseline (control) for statistical analyses.

**Figure 4:**
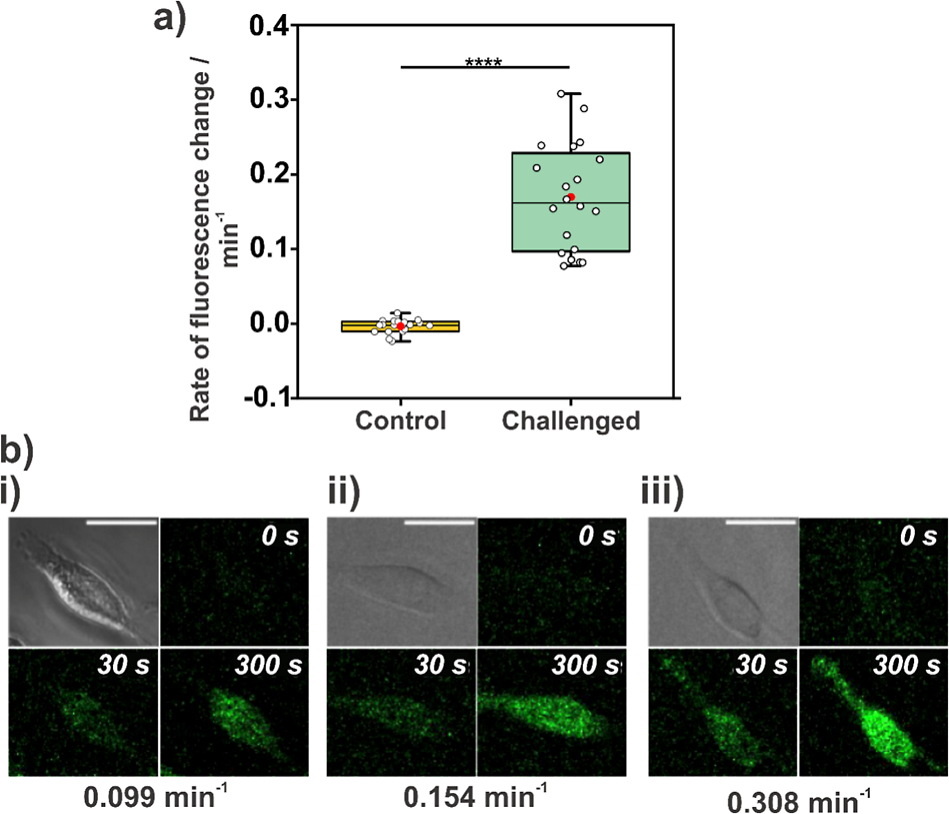
(a) Boxplot to highlight rates of fluorescence change in the control and challenged data sets. (b) Brightfield (top left of quadrant) and fluorescence images of three target cells captured at time points of 0, 30, and 300 s with the respective rates of fluorescence change at the bottom of the image quadrant. Cells represent the smallest (i), average (ii), and largest (iii) rates of change. In panel (a) box size: mean ± 1 SD; whiskers range: 5%–95%; black horizontal line in box center represents median; red dot represents mean value; data points indicated by white circles; **** represents *P* < 0.0001. Scale bar in (b) is 25 μm.

Figure 4a reveals that the physical presence of the microelectrode has minimal impact on the intracellular oxygen level, as indicated by the near-zero rate of fluorescence change. This is expected as the cellular oxygen flux, resulting from respiration, is very small.^35^ The average rate of change for the challenged data was 0.169 ± 0.072 min^–1^, a significant increase relative to the control (−0.003 ± 0.009 min^–1^). All rates of change are derived from the fluorescence data in Tables S4 and S5 (Supporting Information section SI-5). The effect of the galvanostatic challenge in changing the intracellular oxygen levels is further confirmed by a paired *t* test between the challenged and control data (*P* = 4.81 × 10^–9^).

The control data display a narrow, near-zero distribution, highlighting that the microelectrode presence does not disturb cell respiration or local oxygen concentrations. The challenged data group has a broader distribution, as depicted in Figure 4a. The variance of the control data is 7.98 × 10^–5^ min^–2^, which is much smaller than the variance for the challenged cells, 4.71 × 10^–3^ min^–2^. An *F*-test reveals that the variances are indeed different, with *F* > *F* critical (64.4 vs 2.2). Figure 4b portrays the brightfield image and fluorescence images captured during the oxygen challenge for the cells with the smallest, average, and largest rate of changes during the challenge. We also note that minimal photobleaching was observed, as slightly negative fluorescence rates were recorded for some control cells. Conversely, following the galvanostatic challenge, a continuous increase in fluorescence was observed over time, even after a total of 10 min of laser exposure, highlighting the robustness of the hypoxia dye when activated at lower oxygen levels.

Cell area was used as a proxy for intracellular dye concentration, and no correlation between cell size and rate of fluorescence change was observed (Supporting Information Figure S4; Section SI-6), indicating that the distribution in the rate values is not due to different dye uptake or kinetics. Considering that all cells were subject to the same oxygen challenge, i.e., the same local oxygen concentration, the differences in the rate of fluorescence change are therefore most likely from intrinsic differences in the behavior of individual cells and natural heterogeneity within a population, such as different metabolic status and health.

**Figure 5:**
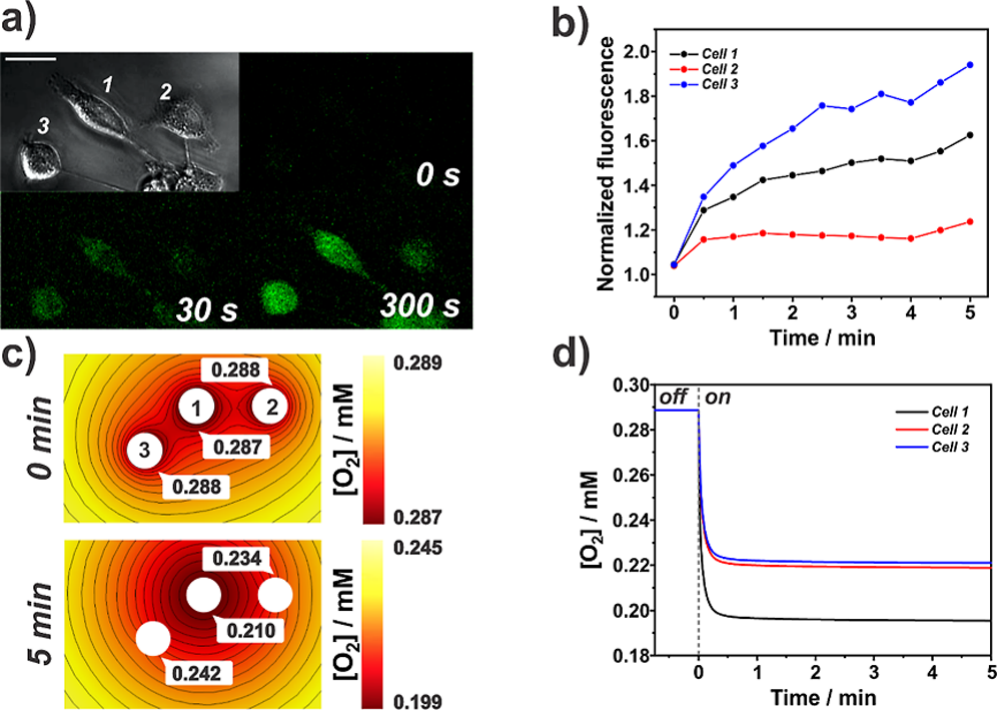
(a) Brightfield image (top left quadrant) and fluorescence images from a time-lapse of a group of 3 cells under the oxygen challenge. The microelectrode was positioned 25 μm from the top of cell 1. (b) Normalized fluorescence over time for the 3 cells in panel (a). (c) Simulated oxygen concentration distribution around the cells before (0 min) and 5 min into the galvanostatic challenge (−7 nA). (d) Simulated average oxygen concentration at the cells over time. Scale bar in panel (a) is 25 μm.

Further evidence of heterogeneous cell behavior is seen when we expand our analyses to neighboring cells of the target cell. The confluency to which the cells were plated (30% to 50%) meant that most cells were in groups. Figure 5a shows a brightfield image and fluorescence images captured at different time points — 0, 30, 300 s — during the galvanostatic challenge (−7 nA) for a group of three cells. The microelectrode was positioned 25 μm above cell 1 (target cell). The fluorescence images in Figure 5a reveal a marked increase in fluorescence intensity over time for all cells, demonstrating that the neighboring cells were also exposed to lower oxygen levels. This is expected as the oxygen depletion layer extends a few microelectrode radii into the solution during the ORR pulse (Figure 2). From the fluorescence intensity-time profile for all three cells (Figure 5b), we can calculate rates of fluorescence change of 0.099, 0.022, and 0.178 min^–1^ for cells 1, 2, and 3, respectively.

Figure 5c shows the simulated oxygen concentration spatial distribution around the cells at 0 and 5 min of challenge. Cell 1 (target cell) is exposed to the lowest oxygen concentration, with cells 2 and 3, at similar radial distances from cell 1, experiencing similar oxygen levels. The simulated extracellular O_2_ concentration around each cell (Figure 5d) does not match the changes in intracellular O_2_ reported by the rates of fluorescence change. Again, no clear correlation between cell area (a proxy for dye concentration) and rate of fluorescence change is seen for this experiment (Figure S5), suggesting that the mismatch between extracellular and reported intracellular oxygen levels is unrelated to the dye. The marked differences in the three cell morphologies indicate cellular health disparities. Cell 3 exhibits a rounded morphology, which is characteristic of poor cell health.^55,56^ This suggests that the mismatch observed could be due to differences in cellular health and metabolism, possibly making cell 3 more susceptible to the oxygen challenge. Prospective studies using this methodology to assess various cell morphologies may elucidate key differences concerning cellular heterogeneity when exposed to the galvanostatic hypoxia challenge.

The ability to observe and measure the effect of the oxygen challenge on single cells within a cell population (Figure 5a) allows for every single cell to be treated as an individual. Coupled with numerical simulations to calculate the oxygen concentration spatial distribution around those cells, our proposed method can provide insights into minute differences in individual behavior within a cell population. This methodology could be further expanded by incorporating dyes to report on specific cell functions, such as mitochondrial function,^57^ and could help to elucidate the origins of the heterogeneous cell response to the oxygen challenge. While we explored ORR in platinum, other electrochemical challenges could be imposed on single cells by performing different reactions at the microelectrode. An intriguing alternative could involve performing ORR over a different electrode material that favors a 2-electron pathway, producing H_2_O_2_^58^ and allowing for the investigation of H_2_O_2_ profiling and the metabolism of various types of cancer cells.^59,60^

## 4 Conclusion

We have demonstrated the use of galvanostatic-control of the ORR at a platinum microelectrode to rapidly induce oxygen deprivation while using a commercial fluorescent dye (Image-iT Green hypoxia reagent) and CLSM to measure the effect on individual cells. This forms the basis of a platform that can elucidate minute differences in cellular behavior at the single-cell level, even within a population. The galvanostatic challenge was able to reduce local oxygen levels in a controlled manner, predicted by numerical simulations, inducing hypoxic conditions to target cells. The oxygen deprivation effects were indicated by a fluorescent assay for which there was a marked increase in the average rate of fluorescence change of the challenged cells relative to the control (unperturbed cells).

The reported intracellular oxygen level of cells subjected to the same challenge was heterogeneous, noted by the larger variance in the rate of fluorescence change compared to the control data, pointing to heterogeneous cellular behavior or health. Neighboring cells near a targeted cell were affected by the galvanostatic challenge to a smaller degree, as predicted by the simulations, allowing for multiple cells within the CLSM field of view to be regarded as individual entities.

This platform provides exciting future scope for studying cellular functions and behaviors of multiple cell lines and tissues under transient oxygen deprivation challenges. Furthermore, it could be extended to explore intermittent (periodic) hypoxia challenges, owing to the inherently fast mass transport rate of microscale electrochemical systems.

## Data Availability

The authors confirm that the data supporting the findings of this study are available within the article and its Supporting Information, or upon further request from the corresponding author. COMSOL reports can also be made available upon reasonable request.

## Supporting Information Available

PC12 cell culture confluency and imaging solution composition, Pt microelectrode details, FEM model details, cell viability assay after the galvanostatic challenge, fluorescence intensity and rate for control and challenged cells, and fluorescence rate vs cell area (PDF)
