# Supplementary material for: Transient Single Cell Hypoxia Induced by Localized Galvanostatic Oxygen Challenge
Source: ACS Meas Sci Au. 2025 Apr 1;5(2):234–41. doi: 10.1021/acsmeasuresciau.4c00100 (PMC12006948; doi:10.1021/acsmeasuresciau.4c00100)
Supplement: Supplementary file 1 — tg4c00100_si_001.pdf [file tg4c00100_si_001.pdf]

# Supporting Information

# Transient single cell hypoxia induced by localized galvanostatic oxygen challenge

Marlene H. Hill[1,2], Gabriel N. Meloni[1,4], Bruno G. Frenguelli[3], Patrick R. Unwin[1*]

[1]Department of Chemistry, [2]Molecular Analytical Science Centre for Doctoral Training (MAS CDT), [3]School of Life Sciences, at the University of Warwick, Coventry CV4 7AL, United Kingdom

[4] Institute of Chemistry, Department of Fundamental Chemistry, University of São Paulo, 05508-000 São Paulo, SP, Brazil

*Corresponding author: p.r.unwin@warwick.ac.uk

**Contents**


| | | |
|---|---|---|
| SI-1 | PC12 cell culture confluency and imaging solution composition | S3 |
| SI-2 | Pt microelectrode fabrication and characterization | S4 |
| SI-3 | FEM model details | S5 |
| SI-4 | Cell viability assay after the galvanostatic challenge | S9 |
| SI-5 | Fluorescence intensity and rate for control and challenged cells | S10 |
| SI-6 | Fluorescence rate *vs.* cell area | S12 |
| | References | S13 |

## SI-1 PC12 cell culture confluency and imaging solution composition

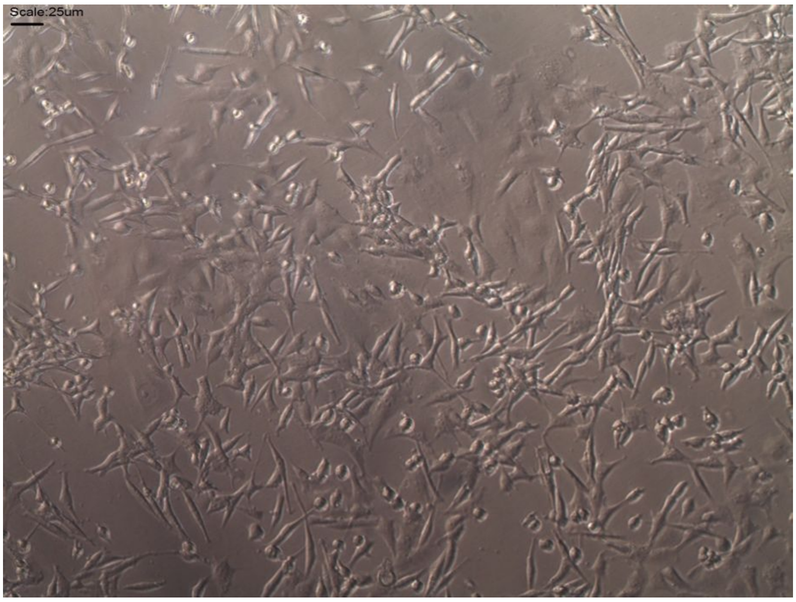


***Figure S1.*** *Example of PC12 cell culture grown in F12K medium in flask prior to splitting. Scale bar represents 25 µm.*

***Table S1.*** *Live Cell Imaging medium composition.*

| **Component** | **Concentration (mM)** |
|---|---|
| HEPES buffer | 20 |
| NaCl | 140 |
| KCl | 2.5 |
| $CaCl_2$ | 1.8 |
| $MgCl_2$ | 1.0 |

## SI-2 Pt microelectrode fabrication and characterization

The disk-shaped Pt microelectrode with a diameter of 25 µm was fabricated[1] by encapsulating a Pt wire with 25 µm diameter (Goodfellow, 99.99%) into a tapered borosilicate glass capillary, which was heated under vacuum, by placing it in the center of a heating coil on a Narishige pipette puller (PC-100). After sealing, the tapered end was polished to create a smooth, flat surface. The diameter of the glass support at the end was 350 µm. The electrodes and overall dimensions were determined by optical microscopy.

## SI-3 FEM model details

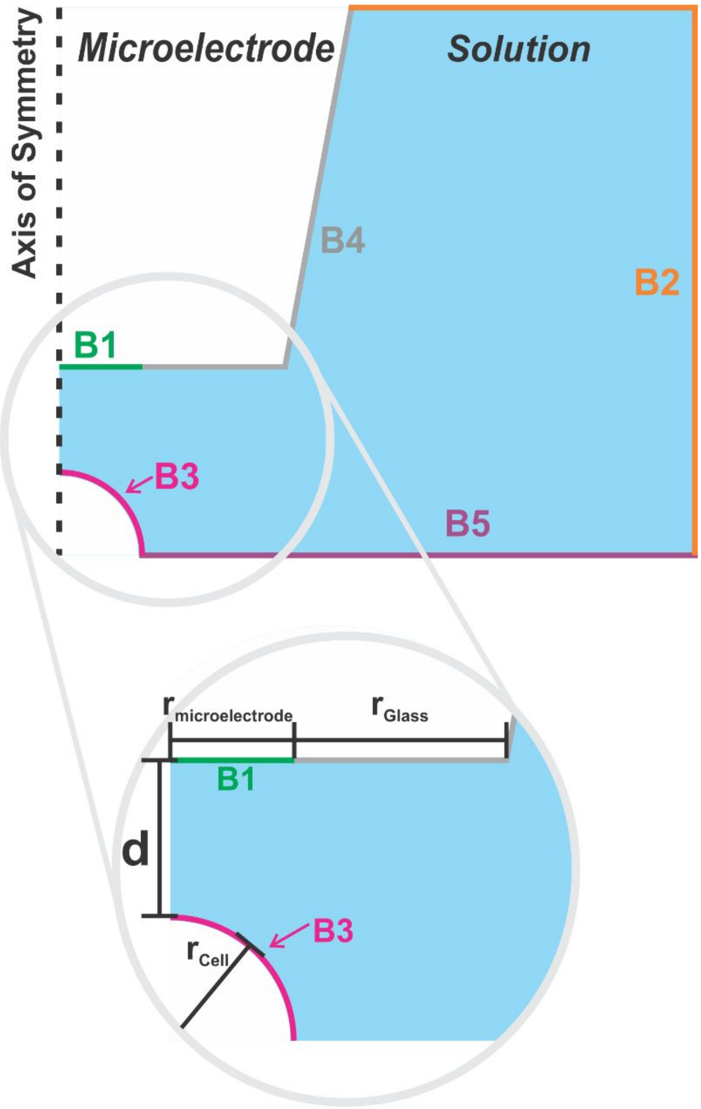


***Figure S2.*** *Schematic illustration of the simulation domain used for the FEM model. Boundary conditions are specified in Table S2. Drawing not to scale.*

All numerical simulations were performed using COMSOL Multiphysics v5.6 and the transport of diluted species module. Simulation of the oxygen concentration profiles around the microelectrode, held at a fixed distance ***d*** (25 μm) from the target and neighbors' cells, was simulated using a 3D domain capturing the individual cell dimensions and cell position relative to the microelectrode. The microelectrode dimensions ($r_{microelectrode}$ and $r_{Glass}$) were deduced from optical microscopy images and electrochemical characterization. The cell geometry was

simplified to a spherical cap of radius $\boldsymbol{r_{Cell}}$, approximated to be half of the largest diameter of a cell measured by optical microscopy. Simulations of proton concentration profiles were performed in a 2D axisymmetric domain considering the microelectrode geometry and a single target cell to reduce computational cost. For both models, mesh density was refined until the results were independent of mesh size. Figure S2 depicts a 2D representation of the simulation domains, highlighting the important geometric aspects and boundary conditions summarized in Table S2.

***Table S2.*** *Summarized boundary conditions for the FEM model. Only fluxes normal to the boundary were considered, noted by "$\boldsymbol{n}$".*

| ***Boundary*** | ***Flux(J) OR Concentration condition*** |
|---|---|
| B1 | $\boldsymbol{n} \cdot J_{O_2} = -\frac{i_{Galvo}}{n\,F\,\pi\,r_{microelectrode}{}^2}$ OR $[O_2] = 0$ M, and $\boldsymbol{n} \cdot J_{H^+} = -4\frac{i_{Galvo}}{n\,F\,\pi\,r_{microelectrode}{}^2}$ |
| B2 | $[O_2]_{Bulk}$ , $[HEPES]_{Bulk}$ |
| B3 | $\boldsymbol{n} \cdot J_{O_2} = -\frac{OCR_{\text{Target cell}}}{2\pi r_{Cell}}$ |
| B4 and B5 | $\boldsymbol{n} \cdot J_{O_2} = 0$ |

All simulations were time-dependent to capture the temporal aspect of the oxygen challenge. For all simulations, the flux of each species *i* is described by the Nernst-Plank equation, considering only diffusion (Equation 1), with continuity equation (Equation 2), where $D_j$ is the diffusion coefficient of the species, and $C_j$ is its concentration.

$$J_i = -D_i \nabla C_i \qquad (1)$$

$$\frac{\partial C_i}{\partial t} + \nabla \cdot J_i = 0 \qquad (2)$$

For the proton concentration simulations, six species were considered: $H_2O$, $OH^-$, $H^+$, HEPES, $HEPES^+$, and $EPES^-$ with the last three representing the neutral, protonated, and deprotonated species in the HEPES buffer, respectively. The reaction between the species at the solution domain is defined by the following rate laws derived from chemical equilibrium:

$$Rate_{OH} = FR \times (K_w - [H^+][OH^-])$$

$$Rate_H = FR \times (K_{a_{HEPES}} \times [HEPES] - [HEPES^-][H^+] + (1000 \times FR) \times (K_{a_{HEPES^+}} \times [HEPES^+] - [HEPES][H^+]) + FR \times (K_w - [H^+][OH^-])$$

$$Rate_{HEPES^+} = -FR \times (K_{a_{HEPES^+}} \times [HEPES^+] - [HEPES][H^+]$$

$$Rate_{HEPES} = -FR \times (K_{a_{HEPES}} \times [HEPES] - [EPES^-][H^+]) + FR \times (K_{a_{HEPES^+}} \times [HEPES^+] - [HEPES][H^+]$$

$$Rate_{HEPES^-} = FR \times (K_{a_{HEPES}} \times [HEPES] - [HEPES^-][H^+])$$

Table S3 contains the concentrations, diffusion coefficients, and dissociation constants used for all simulations. Note that FR was capped at a low value to ensure that simulations converged, but it was sufficiently large to allow the reactions to approach close to equilibrium within the mesh of the simulation domain. As noted in the main text, pH changes under buffered conditions are minimal in any case, and the pH results should be considered illustrative.

*__Table S3.__ List of physicochemical parameters used for the simulations.*

| **Symbol** | **Value** | **Description** |
|---|---|---|
| $[O_2]_{Bulk}$ | 289 μM | Oxygen concentration at bulk solution[2] |
| $[HEPES]_{Bulk}$ | $\frac{[HEPES^-] \times [H^+]}{K_{a_{HEPES}}}$ | HEPES concentration at bulk solution |
| $[HEPES^+]_{Bulk}$ | $\frac{[HEPES] \times [H^+]}{K_{a_{HEPES+}}}$ | $HEPES^+$ concentration at bulk solution |
| $[HEPES^-]_{Bulk}$ | 20 mM | $HEPES^-$ concentration at bulk solution |
| $[H^+]_{Bulk}$ | $10^{-pH}$ | Proton concentration at bulk |
| $[OH^-]_{Bulk}$ | $10^{-(pKw-pH)}$ | Hydroxide concentration at bulk |
| $D_{HEPES, HEPES^+, EPES^-}$ | $5 \times 10^{-6}$ cm$^2$ s$^{-1}$ [3] | Diffusion coefficient of HEPES components |
| $D_{H^+}$ | $9.3 \times 10^{-5}$ cm$^2$ s$^{-1}$ | Diffusion coefficient of protons |
| $D_{OH^-}$ | $5.3 \times 10^{-5}$ cm$^2$ s$^{-1}$ | Diffusion coefficient of hydroxide |
| $D_{O_2 Solution}$ | $2.2 \times 10^{-5}$ cm$^2$ s$^{-1}$ [4] | Diffusion coefficient oxygen in solution |
| $pK_{aHEPES}$ | 3 | -log of the first acid dissociation constant of HEPES |
| $pK_{aHEPES+}$ | 7.56 | -log of the second acid dissociation constant of HEPES |
| $OCR_{Target\ cell}$ | 2.15 pmol s$^{-1}$ | Target cell oxygen consumption rate [5] |
| FR | $1 \times 10^6$ m$^3$ s$^{-1}$mol$^{-1}$ | Forward rate constant for the rate laws |

Oxygen reduction currents ($i_{Galvo}$, Table S2, B1) for the galvanostatic challenges were converted to molecular flux assuming a 4 electron, 4 proton, reduction process at the microelectrode surface. Oxygen and proton concentrations over the cell wall were calculated by averaging the concentration at the cell/solution boundary (B3 in Figure S2). For the oxygen concentration simulations, a constant inward flux of oxygen, representing cell respiration, was applied to the cell boundary B3 (Table S2). A first stationary simulation step was performed with $i_{Galvo}$ equal to 0 nA, setting the initial oxygen concentration for the time-dependent step.

## SI-4 Cell viability assay after the galvanostatic challenge

**a)**

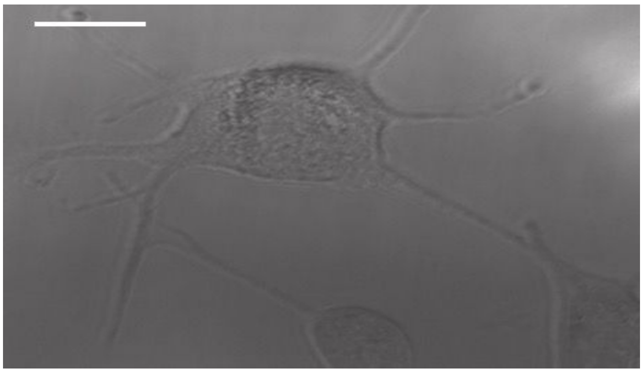

**b)**

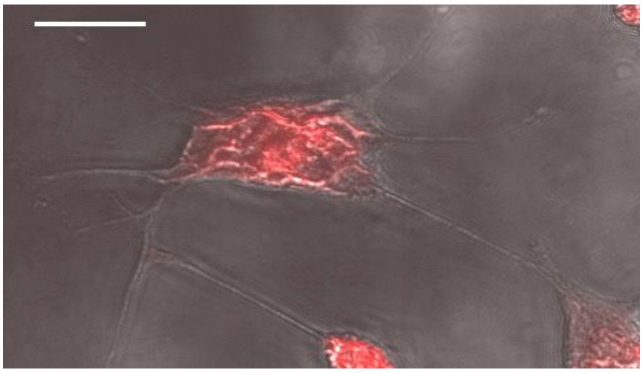

***Figure S3.*** *Composite image of a challenged cell after 10 minutes of PI staining (a). Composite image of the same cell after the addition of 10% DMSO (b). Fluorescence images were recorded with an excitation wavelength of 535 nm and an emission wavelength range of 613-621 nm. Scale bar represents 25 µm.*

To confirm if target cells exposed to the oxygen challenge were still viable, experiments were performed with cells subjected for 5 minutes to an ORR current of -7 nA. 4.5 µM propidium iodide (PI) dye, an indicator of cell viability and death, was added after the experiments. The excitation wavelength of PI is close to the emission of the hypoxia dye, so cells could not be co-stained with both dyes. Figure S3a shows the challenged cell's PI fluorescence after 10 minutes of staining overlaid with a brightfield image, and no fluorescence is observed. Figure S3b shows the positive control test, where 10 % DMSO was added to the solution, inducing cell death, indicated by the PI fluorescence, colored in red.

## SI-5 Fluorescence intensity and rate for control and challenged cells

*Table S4. Raw fluorescence intensity data of all target cells challenged for five minutes captured at 30 s intervals*

| Time / min | Cell A | Cell B | Cell C | Cell D | Cell E | Cell F | Cell G | Cell H | Cell J |
|---|---|---|---|---|---|---|---|---|---|
| **0** | 1.428 | 1.245 | 1.392 | 1.303 | 1.355 | 1.217 | 1.198 | 1.237 | 1.284 |
| **0.5** | 1.467 | 1.303 | 1.522 | 1.345 | 1.392 | 1.499 | 1.480 | 1.533 | 1.469 |
| **1** | 1.477 | 1.385 | 1.599 | 1.379 | 1.423 | 1.568 | 1.640 | 1.643 | 1.583 |
| **1.5** | 1.533 | 1.454 | 1.685 | 1.395 | 1.429 | 1.657 | 1.689 | 1.771 | 1.690 |
| **2** | 1.600 | 1.530 | 1.785 | 1.476 | 1.493 | 1.682 | 1.791 | 1.838 | 1.773 |
| **2.5** | 1.664 | 1.618 | 1.846 | 1.525 | 1.584 | 1.704 | 1.896 | 1.906 | 1.826 |
| **3** | 1.701 | 1.693 | 1.927 | 1.570 | 1.570 | 1.747 | 2.020 | 1.902 | 1.892 |
| **3.5** | 1.772 | 1.818 | 2.054 | 1.571 | 1.595 | 1.768 | 2.133 | 2.044 | 1.931 |
| **4** | 1.886 | 1.936 | 2.138 | 1.632 | 1.675 | 1.756 | 2.288 | 2.207 | 1.990 |
| **4.5** | 1.926 | 2.111 | 2.271 | 1.641 | 1.726 | 1.807 | 2.405 | 2.319 | 2.060 |
| **5** | 2.002 | 2.211 | 2.313 | 1.676 | 1.793 | 1.892 | 2.516 | 2.439 | 2.141 |

| Time / min | Cell K | Cell L | Cell M | Cell N | Cell O | Cell P | Cell Q | Cell R | Cell S | Cell T |
|---|---|---|---|---|---|---|---|---|---|---|
| **0** | 1.239 | 1.441 | 1.592 | 1.391 | 1.504 | 1.614 | 1.766 | 1.115 | 1.146 | 1.434 |
| **0.5** | 1.382 | 1.621 | 1.748 | 1.389 | 1.733 | 1.825 | 1.967 | 1.198 | 1.350 | 1.749 |
| **1** | 1.459 | 1.754 | 1.966 | 1.452 | 1.872 | 1.901 | 2.043 | 1.256 | 1.442 | 1.877 |
| **1.5** | 1.468 | 1.889 | 2.178 | 1.496 | 2.024 | 2.018 | 2.112 | 1.301 | 1.600 | 2.032 |
| **2** | 1.565 | 1.941 | 2.334 | 1.551 | 2.149 | 2.082 | 2.182 | 1.360 | 1.695 | 2.135 |
| **2.5** | 1.663 | 2.098 | 2.434 | 1.578 | 2.342 | 2.169 | 2.310 | 1.415 | 1.789 | 2.234 |
| **3** | 1.696 | 2.255 | 2.648 | 1.604 | 2.469 | 2.249 | 2.437 | 1.459 | 1.936 | 2.367 |
| **3.5** | 1.771 | 2.294 | 2.736 | 1.660 | 2.633 | 2.299 | 2.442 | 1.482 | 2.023 | 2.455 |
| **4** | 1.868 | 2.446 | 2.953 | 1.707 | 2.748 | 2.347 | 2.529 | 1.506 | 2.096 | 2.531 |
| **4.5** | 1.966 | 2.545 | 2.911 | 1.729 | 2.864 | 2.420 | 2.562 | 1.591 | 2.176 | 2.591 |
| **5** | 2.034 | 2.683 | 3.170 | 1.791 | 2.935 | 2.455 | 2.643 | 1.597 | 2.294 | 2.764 |

***Table S5.*** *Table to show obtained fluorescence rates for both control and challenged conditions. The number indicates the cell dish.*

| Cell | Control / a.u min$^{-1}$ | Test / a.u. min$^{-1}$ |
|---|---|---|
| A_1 | -0.010 | 0.119 |
| B_1 | -0.007 | 0.193 |
| C_1 | -0.024 | 0.184 |
| D_1 | -0.002 | 0.077 |
| E_1 | -0.011 | 0.085 |
| F_2 | 0.014 | 0.099 |
| G_2 | 0.004 | 0.243 |
| H_2 | 0.003 | 0.208 |
| I_2 | N/A | 0.082 |
| J_2 | -0.002 | 0.154 |
| K_2 | 0.001 | 0.150 |
| L_2 | 0.003 | 0.238 |
| M_2 | -0.002 | 0.308 |
| N_2 | -0.002 | 0.082 |
| O_2 | 0.005 | 0.288 |
| P_3 | -0.021 | 0.157 |
| Q_3 | -0.011 | 0.166 |
| R_4 | -0.003 | 0.095 |
| S_4 | 0.002 | 0.220 |
| T_4 | -0.002 | 0.237 |

## SI-6 Fluorescence rate *vs.* cell area

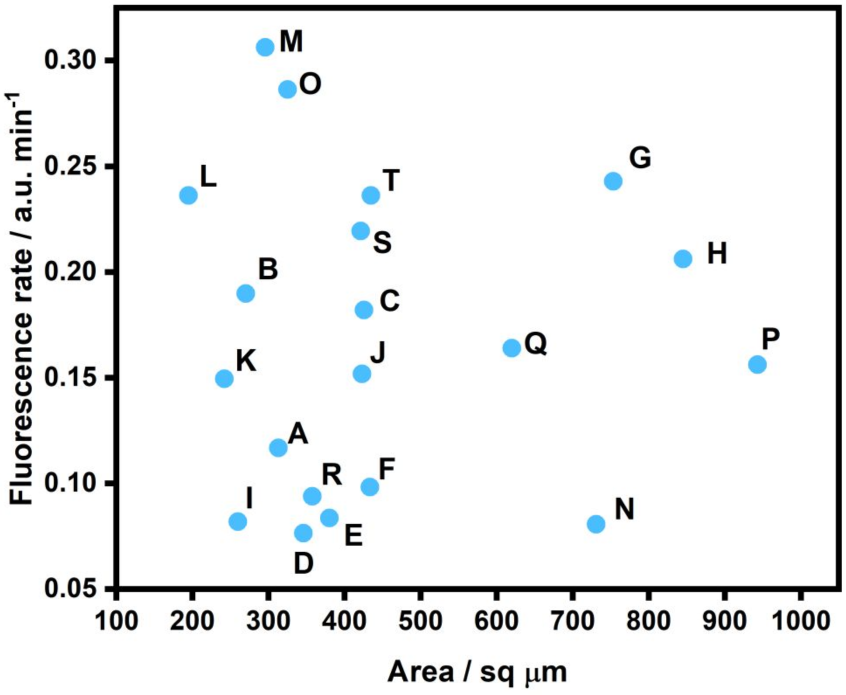


***Figure S4.*** *Graph to show fluorescence rate vs. cell area of labeled challenged cells.*

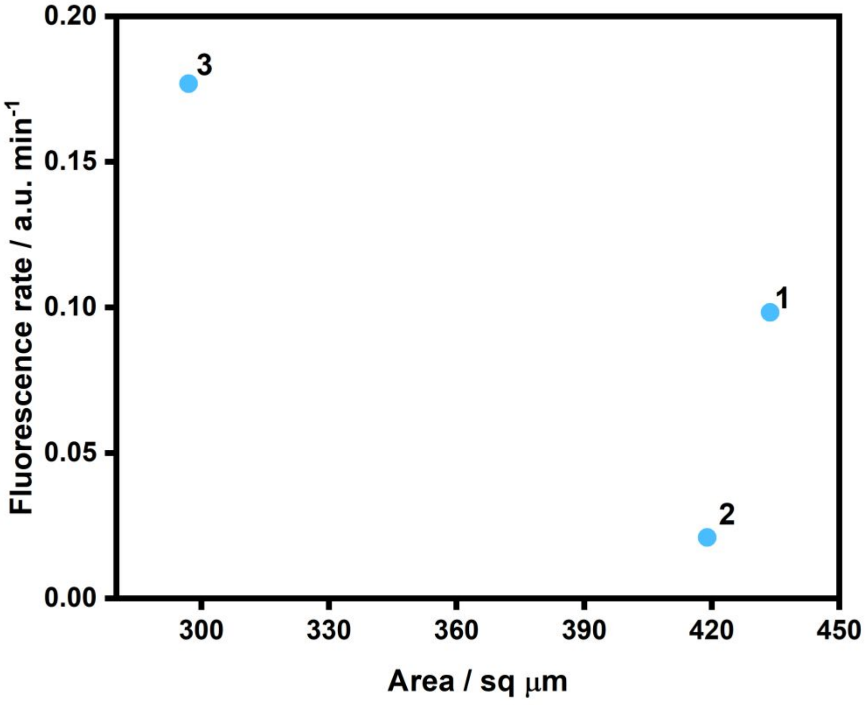


***Figure S5.*** *Graph to show fluorescence rate vs. cell area of cells in Figure 5a.*

## References


(1) Bard, A. J.; Fan, F. R. F.; Kwak, J.; Lev, O. Scanning Electrochemical Microscopy. Introduction and Principles. *Anal. Chem.* **1989**, *61* (2), 132–138. https://doi.org/10.1021/ac00177a011.

(2) Sharma, S.; Hashmi, M. F. *Partial Pressure Of Oxygen*; StatPearls Publishing, 2022.

(3) Saparov, S. M.; Antonenko, Y. N.; Pohl, P. A New Model of Weak Acid Permeation through Membranes Revisited: Does Overton Still Rule? *Biophys. J.* **2006**, *90* (11), L86–L88. https://doi.org/10.1529/biophysj.106.084343.

(4) Han, P.; Bartels, D. M. Temperature Dependence of Oxygen Diffusion in H 2 O and D 2 O †. *J. Phys. Chem.* **1996**, *100* (13), 5597–5602. https://doi.org/10.1021/jp952903y.

(5) Im, A.-R.; Chae, S.-W.; Zhang, G. jun; Lee, M.-Y. Neuroprotective Effects of Psoralea Corylifolia Linn Seed Extracts on Mitochondrial Dysfunction Induced by 3-Nitropropionic Acid. *BMC Complement. Altern. Med.* **2014**, *14* (1), 370. https://doi.org/10.1186/1472-6882-14-370.
